# Spectral Phase Shift Interferometry for Refractive Index Monitoring in Micro-Capillaries

**DOI:** 10.3390/s20041043

**Published:** 2020-02-14

**Authors:** Valentina Bello, Alberto Simoni, Sabina Merlo

**Affiliations:** Department of Electrical, Computer and Biomedical Engineering, University of Pavia, 27100 Pavia, Italy; alberto.simoni01@universitadipavia.it (A.S.); merlo@unipv.it (S.M.)

**Keywords:** rectangular glass micro-capillaries, optical resonators, optical interferometry, phase detection, micro-opto-fluidic, optical sensing

## Abstract

In this work, we demonstrate spectral phase-shift interferometry operating in the near-infrared wavelength range for refractive index (RI) monitoring of fluidic samples in micro-capillaries. A detailed theoretical model was developed to calculate the phase-sensitive spectral reflectivity when low-cost rectangular glass micro-capillaries, filled with samples with different refractive indices, are placed at the end of the measurment arm of a Michelson interferometer. From the phase-sensitive spectral reflectivity, we recovered the cosine-shaped interferometric signal as a function of the wavelength, as well as its dependence on the sample RI. Using the readout radiation provided by a 40-nm wideband light source with a flat emission spectrum centered at 1.55 µm and a 2 × 1 fiberoptic coupler on the common input-output optical path, experimental results were found to be in good agreement with the expected theoretical behavior. The shift of the micro-capillary optical resonances, induced by RI variations in the filling fluids (comparing saline solution with respect to distilled water, and isopropanol with respect to ethanol) were clearly detected by monitoring the positions of steep phase jumps in the cosine-shaped interferometric signal recorded as a function of the wavelength. By adding a few optical components to the instrumental configuration previously demonstrated for the spectral amplitude detection of resonances, we achieved phase-sensitive detection of the wavelength positions of the resonances as a function of the filling fluid RI. The main advantage consists of recovering RI variations by detecting the wavelength shift of “sharp peaks”, with any amplitude above a threshold in the interferometric signal derivative, instead of “wide minima” in the reflected power spectra, which are more easily affected by uncertainties due to amplitude fluctuations.

## 1. Introduction

In the last few years, researchers have strongly focused on the investigation of optical sensors for the measurement of the refractive index (RI) of liquid substances in view of (bio)chemical analyses. RI detection, although not specific, is greatly appreciated and widely pursued since it achieves label-free sensing, without adding exogenous markers and affecting the intrinsic characteristics of the sample. Moreover, several optical readout methods have been reported that allow remote and contactless analyses, being thus minimally invasive techniques, an important feature to bear in mind, particularly when dealing with biological fluids. In the scientific literature, many sensors addressing these requirements have been investigated, featuring resonant micro-cavities [1,2,3,4], ring resonators [5,6,7], photonic crystals [8,9,10,11,12,13] or whispering gallery modes [14]. However, the proposed solutions are mainly based on expensive custom-designed devices that require complex micro-machining facilities. 

Rectangular glass micro-capillaries are low-cost miniaturized devices, commercially available in several formats. They can be exploited for the optical sensing of fluid samples, thanks to their interesting features: indeed, they allow a remote non-contact, non-invasive analysis of ultra-small volumes of quantities, of the order of a few μL or even nL. Moreover, their symmetric structure and flat surface strongly reduce the issues related to light scattering that arise when using round-section capillaries. As reported in previous works [15,16,17], rectangular micro-capillaries can be envisioned as optical resonators: when they are illuminated by broadband light, the reflected power spectrum exhibits a sequence of minima characterized by a wavelength position that depends on the geometrical parameters of the micro-device and the material filling the channel. In particular, when the RI of the sample increases, the optical resonances shift towards higher wavelengths. Hence, by monitoring the wavelength shift, it was possible to recover the variation of the RI with respect to a reference fluid. Moreover, thanks to the transparency of the micro-capillary in the near-infrared region, a more sophisticated instrumental configuration was implemented to collect both the spectral reflectivity *R*(*λ*) and transmissivity *T*(*λ*) [16]. The *T*(*λ*)/*R*(*λ*) ratio was calculated, leading to a spectrum characterized by narrow and sharp peaks located at the micro-capillary resonance wavelengths. Computing the ratio allows compensating for fluctuations in the power emitted by the source, improving the dynamic range of the sensor and detecting the resonance positions on narrow peaks, but with the drawback of a more complicated setup.

Optical resonances can be identified in reflection by using a different method based on spectral phase interferometry. This technique was exploited for the first time in 1996 to detect the surface plasmon resonances (SPR) of SPR-based sensors [18]: the measurement was based on the analysis of the relative phase shift between the TE- and TM-polarized components of the electric field. This phase method has been widely employed both in prism-based [19] and fiber-based [20,21] SPR sensors for monitoring refractive index variations occurring on the sensor surface.

In this work, we exploit spectral phase-shift interferometry for monitoring volume RI variations in liquid samples filling the micro-capillary channel. First, we present the theoretical model we developed to calculate the behavior of the interferometric signal in the wavelength domain when the capillary is inserted along the measurement arm of a Michelson interferometer. Theoretical results are provided by supposing that the channel was filled with water and saline solution. Experimental measurements were carried out by applying the radiation emitted by a fiberoptic-coupled 40-nm broadband light source, centered at a wavelength of 1.55 μm, and collecting the reflected power spectra as well as interferometric signals, as a function of the wavelength, with an optical spectrum analyzer (OSA). The air–glass interface of a glass slab plays the role of a beam-splitter to direct the readout radiation partially along the measurement arm (ending with a micro-capillary) and partially, at 90°, along the reference arm (ending with an Al-coated mirror) of a Michelson interferometer as well as to recombine and redirect the reflected fields toward the OSA. A 2x1 single-mode fiberoptic coupler with a 50:50 splitting ratio terminated with an aspherical lens allows us to separate, from the input light, the reflected contributions at the output.

The shift of the optical resonances of standard, low-cost glass micro-structures, induced by RI variations in the filling fluids (comparing saline solution with respect to distilled water, and isopropanol with respect to ethanol), has been detected by monitoring the positions of the steep amplitude jumps, due to abrupt phase changes, appearing in the cosine-shaped interferometric signal recorded as a function of the wavelength. By adding a few optical components to the instrumental configuration previously demonstrated for the spectral amplitude detection of resonances, we have achieved phase-sensitive detection of the wavelength positions of the resonances as a function of the filling fluid RI. The main advantage consists in recovering RI variations by detecting the wavelength shift of “sharp peaks”, with any amplitude above a threshold in the interferometric signal derivative, instead of “wide minima” in the reflected power spectra, more easily affected by uncertainties due to amplitude fluctuations. To our knowledge, this is the first time that spectral phase interferometry has been used to detect the wavelength shift of micro-opto-fluidic device resonances for monitoring RI variations.

## 2. Rectangular Micro-Capillary Structure

**Figure 1:**
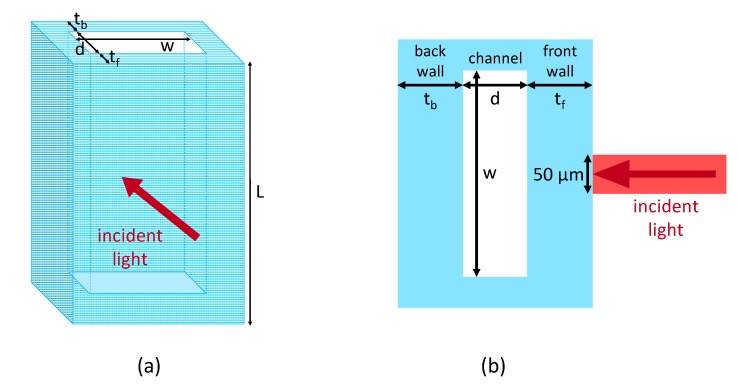
(**a**) Schematic structure of the rectangular glass micro-capillary. (**b**) Top view of the device.

The core devices of the proposed micro-opto-fluidic sensing platform are standard rectangular hollow micro-capillaries (VitroTubes^TM^, VitroCom, NJ, USA), composed of borosilicate glass with a refractive index of about 1.5 at 1550 nm (Figure 1) [22]. Each micro-capillary is constituted by three different layers, consisting of front and back glass walls separated by the inner channel. The device investigated in this work (Vitrocom, VitroTubes #5015) is characterized by the following nominal dimensions: thickness *t_f_* and *t_b_* of the walls and depth *d* of the channel *t_f_* = *d* = *t_b_* = 50 μm, channel width *w* = 1 mm and length *L* = 5 cm. The manufacturer reports a tolerance of 10% for channel depth and of 20% for wall thicknesses. As reported in Figure 1, the readout light beam is shone orthogonally to the flat side of the capillary and it is back-reflected along the same direction due to the refractive index mismatch among the various layers. The diameter of the light spot incident onto the capillary surface is equal to 50 µm (Figure 1b).

## 3. Theoretical Analysis

The micro-capillary can be envisioned as an optical resonator constituted by a sequence of three Fabry–Pèrot etalons composed by materials with different refractive indices: the front wall, the channel and the back wall. At each interface between layers with different refractive index, light is partially reflected and partially transmitted. In previous works [15,16,17], the overall theoretical spectral reflectivity *R*(*λ*) and transmissivity *T*(*λ*) as a function of the wavelength *λ* were retrieved by recursively applying the Fresnel formulas to calculate the electric field traveling back and forth and the spectral power density of reflected and transmitted signals. In particular, the spectral reflectivity *R*(*λ*) is given by:

*R*(*λ*) = |*r_tot_*(*λ*)|^2^, (1)

where *r_tot_*(*λ*), the electric field reflection coefficient of the whole structure, is a complex function of the wavelength. In this work, we have further enhanced the model to calculate the wavelength dependence of the interferometric signal that is photo-detected when the micro-capillary is placed at the end of the measurement arm of a Michelson interferometer and a mirror is located at the end of the reference arm. The interference between both back-reflected electric fields yields the typical cosine-shaped interferometric signal *I_interf_*(*λ*) [23]

*I_interf_*(*λ*) ∝ cos [*φ_tot_*(*λ*)] = cos [*φ_cap_*(*λ*) + *φ_interf_*(*λ*)], (2)

where the total phase *φ_tot_*(*λ*) is the sum of *φ_cap_*(*λ*), which is the phase of the complex reflection coefficient of the capillary, and *φ_interf_*(*λ*), which is the phase contribution due to the length mismatch *Δs* between the two arms of the interferometer:

*φ_interf_*(*λ*) = 2∙k∙*Δs*, (3)

where *k* = 2∙π/*λ* is the wavevector and *λ* is the wavelength. *φ_cap_*(*λ*) can be retrieved by applying the following formulas:

exp [*i∙φ_cap_*(*λ*)] = *r_tot_*(*λ*)/|*r_tot_*(*λ*)|, (4)

*φ_cap_*(*λ*) = Im {ln [*r_tot_*(*λ*)/|*r_tot_*(*λ*)|]}, (5)

where |∙| denotes the modulus, *Im* indicates the imaginary part of a complex number and *ln* is the natural logarithm.

## 4. Theoretical Results

**Figure 2:**
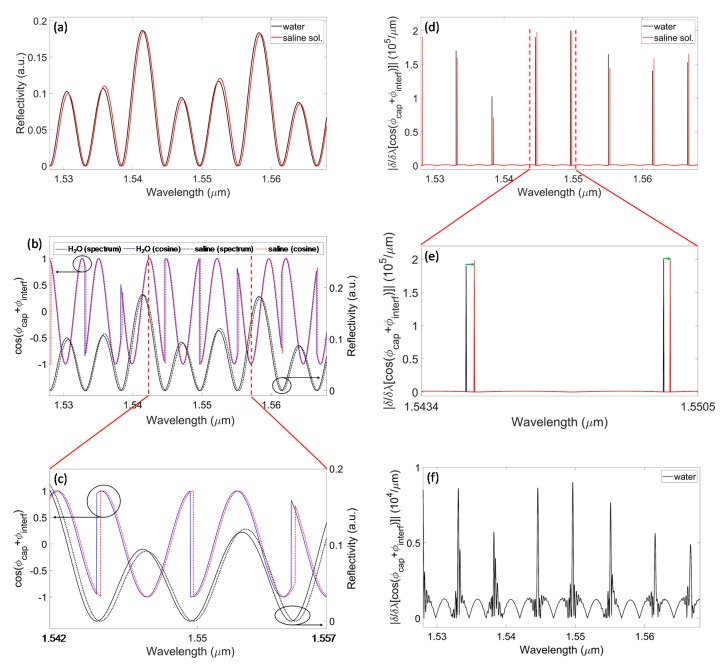
Theoretical results for a capillary with *t_f_* = *d* = *t_b_* = 50 μm. (**a**) Spectral reflectivity *R_tot_*(*λ*) of the capillary, simulating water (black trace) and saline solution (red trace) as fluids filling the channel. (**b**) Cosine-like signals retrieved from Equation 2 for the capillary filled with water (blue solid trace) or saline solution (red dotted trace), compared with the spectral reflectivity (black solid trace: water; black dotted trace: saline solution): steep amplitude changes in the interferometric signals are located in correspondence of the spectral minima, as it can be better observed in the zoomed view (**c**). (**d**) Absolute value of the derivative of the cosine signals with respect to the wavelength (filling fluids: water, black trace; saline solution, red trace). (**e**) Details of the derivative in a narrow spectral range. The green arrows show the spectral shift from water to saline solution. (**f**) Absolute value of the derivative computed after adding white Gaussian noise to the reflection coefficient of the capillary, in the case of water as filling fluid.

Theoretical analyses were carried out in the near-infrared (NIR) wavelength range 1.528–1.568 μm, considering the micro-capillary with nominal geometrical parameters *t_f_* = *d* = *t_b_* = 50 μm (Figure 2). Simulations were performed by considering the inner channel filled with two fluids, distilled water and saline solution, using the values of 1.3340 and 1.3345, respectively, for the real part of the refractive index. Due to the limited depth of the channel, water absorption was neglected and, thus, the imaginary part of the refractive index was not considered in the simulation. The capillary spectral reflectivity is characterized by minima at wavelengths corresponding to the optical resonances of the multilayer structure. The wavelength positions of resonances depend on the thickness of the layers and, in particular, on the RI of the liquid contained in the channel, as demonstrated in previous work [15,16,17]. Moreover, as shown in Figure 2a, the overall spectrum shifts towards higher wavelengths when the sample RI increases from water (black trace) to saline solution (red trace). As the aim of this work is to move from the spectral amplitude detection, reported in previous publications, to the phase-sensitive detection of the spectral shift, we calculated the cosine-shaped interferometric signals obtained when the capillary, placed in the measurment arm of the Michelson scheme, is filled with different fluids. In Figure 2b, and in the zoomed view of Figure 2c, we show the cosine-shaped signals (given by Equation 2) that result from the phase-sensitive detection obtained when considering water (blue solid trace) and saline solution (red dotted trace) as channel-filling fluids. The cosine signals are compared, in the same figures, with the corresponding reflectivity spectra calculated in the absence of the reference path contribution (black solid trace: water; black dotted trace: saline solution). Here, the value of the length mismatch *Δs* was set equal to 136.7 μm. These graphs highlight that the cosine-shaped interferometric signals exhibit well-defined, sharp amplitude variations, due to phase jumps, at the same wavelengths where spectral reflectivity minima are located. As occurs for the minima in amplitude spectral detection, the wavelength positions of the phase jumps undergo a red-shift when the refractive index of the filling fluid does increase. In order to enhance the steep amplitude variations, for a better recognition, the derivatives with respect to the wavelength of the cosine signals were computed and the absolute values are shown in Figure 2d, with a zoomed view in Figure 2e (the green arrows highlight the spectral shift of the peak position). In order to better model a laboratory experimental situation, the theoretical study was also performed by adding white Gaussian noise to the overall reflection coefficient of the capillary itself: the “noisy” derivative was retrieved and Figure 2f shows the result when the capillary is supposed to be filled with water. It is evident that the noise addition does not impair peak detection. Moreover, we retrieved the theoretical sensitivities of the sensor before and after adding the Gaussian noise. The sensitivity *S* of a resonance is calculated as the induced wavelength shift *Δλ* for a given RI variation *Δn*, *S* = *Δλ*/*Δn*. Considering the theoretical results before adding the noise, *S* varies in the range 295–425 nm/RIU, for the resonances at the considered near-infrared wavelengths, while theoretical sensitivities obtained after adding the noise are in the range 290–440 nm/RIU. Hence, the presence of the noise does not negatively affect the performances of the sensors in terms of its sensitivity. Adding the noise only slightly increases the uncertainty in the wavelength position of the derivative peaks. In addition, the full width at half maximum (FWHM) of the theoretical derivative peaks before and after considering the presence of noise was computed. For the theoretical maxima (reported in Figure 2d) obtained before adding the white Gaussian noise, the FWHMs are limited by the wavelength step (10 pm) used to carry out the simulations. Supposing an ideal sampling with an infinite number of points, the FWHM of each peak would be equal to zero. On the other hand, for the theoretical peaks obtained after adding the noise (Figure 2f), the FWHMs have finite values (due to the noise effects) of the order of 200 pm.

## 5. Experimental Setup

**Figure 3:**
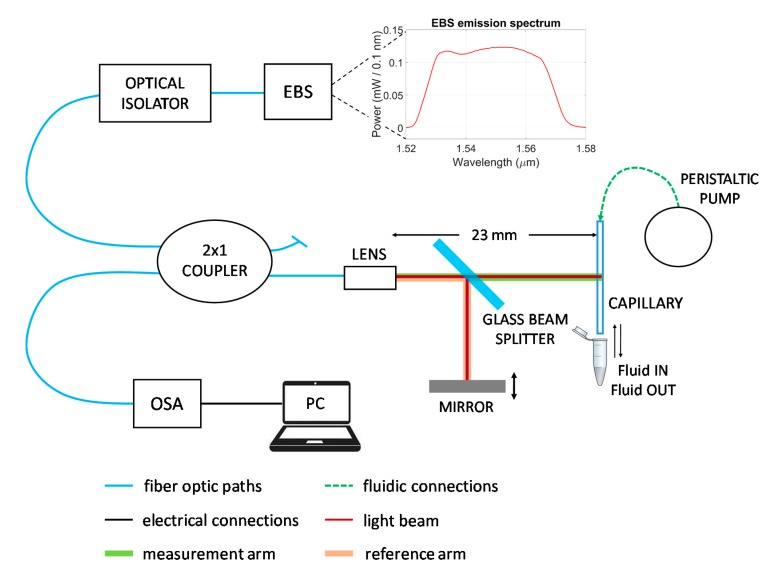
Block diagram of the experimental setup including the Michelson interferometer. Erbium-doped broadband source (EBS). Optical spectrum analyzer (OSA). Personal computer (PC). The graph reports the emission spectrum of the EBS, as coupled into a standard telecom single-mode fiber and measured with the OSA using the resolution bandwidth *RB* = 0.1 nm.

For the experimental verification of the analytical results, phase-sensitive measurements were performed by employing the optical instrumental configuration shown in Figure 3. The micro-capillary is vertically fixed to a metallic frame with a thin layer of glue to improve its stability and, hence, avoid unwanted vibrations of the device. The liquid fills the channel by capillary action and it is discarded after the test by pushing air with a peristaltic pump connected to the upper termination of the micro-capillary. Infrared radiation with an average power density of 0.12 mW/0.1 nm in a wavelength band centered at 1550 nm and a FWHM of 40 nm, is generated by a diode-pumped, Er^3+^-doped fiber broadband source (EBS-4022, MPB Technologies Inc., Canada). The light source is operated in continuous wave mode. The emitted light is coupled into a single-mode optical fiber and guided through an optical isolator, which protects the source from back-reflections, towards a 2x1 fiberoptic coupler with a 50:50 splitting ratio. The output port of the coupler terminates with an aspherical lens (OzOptics, Canada) that provides a low-divergence beam traveling in free-space. The beam reaches a glass slab (with a thickness of 4 mm) tilted at 45°, which acts as a beam splitter. About 96% of the light is transmitted at each interface of the beam splitter. Hence, 92% of the light (96% ∙ 96% ≈ 92%) is transmitted through the glass slab, and then travels along the measurement arm of the Michelson interferometer, reaching and crossing the flat side of the capillary. A small fraction of the radiation hitting the slab (approximately 4%) is reflected by the first glass–air interface at 90°, along the interferometer reference path, towards an Al-coated mirror (ME1S-G01 Thorlabs, NJ, USA) mounted on a precision mechanical stage. The radiation reflected at the back glass–air interface of the glass slab does not reach the mirror and is thus discarded. Light beams back-reflected by the capillary and the mirror are then superposed by the beam splitter and re-collected by the same input lens and fiber. The fiberoptic coupler provides, on the third port, the output radiation that is then coupled to the optical spectrum analyzer (OSA Agilent 86142B, Agilent Technologies, CA, USA), which is connected to a computer for data acquisition.

## 6. Experimental Results

In order to verify the validity of the theoretical analyses and give a proof-of-principle of spectral phase-shift interferometry for refractive index monitoring in micro-capillaries, experimental measurements were performed on a rectangular hollow micro-tube with geometrical parameters *t_f_* = *d* = *t_b_* = 50 μm. It must be noted that the wavelength dependence of the photo-detected interferometric signal is given by the expression

*I_interf exp_*(*λ*) = *I_cap_*(*λ*) + *I_mirror_*(*λ*) + 2∙[ *I_cap_*(*λ*)∙*I_mirror_*(*λ*)]^1/2^∙*V*∙cos [*φ_tot_*(*λ*)], (6)

where *I_cap_*(*λ*) is the signal intensity only due to the capillary reflection, *I_mirror_*(*λ*) is the signal intensity only due to the mirror reflection, *φ_tot_* is the total phase as mentioned in Equation 2 and *V* is the fringe visibility, a parameter that is controlled by the autocorrelation function of the readout light [23]. *V* can be assumed to be equal to one if the unbalance between the interferometer arm lengths *Δs* is much shorter than the coherence length *L_C_* of the photodetected light. *L_C_* can be computed using the formula *L_C_* = *λ_e_*^2^/(*n*∙*Δλ*), where *λ_e_* is the central emission wavelength, *n* = 1 is the RI of air and *Δλ* is the photodetection bandwidth. Since *Δλ* to be considered is the resolution bandwidth of the OSA (0.1 nm), we get *L_C_* = 24 mm; since we are working in a quasi-matched condition, then *L_C_* >> *Δs* and *V* ≈ 1. Signals were acquired by the OSA with a wavelength step of 10 pm in the following sequence. First, *I_cap_*(*λ*) was collected by blocking the reflection coming from the reference mirror. Then, the signal *I_mirror_*(*λ*) was recorded by blocking the signal coming back from the capillary. Finally, the overall interferometric signal *I_interf exp_*(*λ*) with both contributions was collected. During the experiments, from sample to sample, the position of the mirror was mainly kept fixed or just finely modified to obtain the condition *Δs* ≈ 0. After acquisitions, data were processed and the cosine signal was retrieved as

cos [*φ_tot_*(*λ*)] = [*I_interf exp_*(*λ*) – *I_cap_*(*λ*) – *I_mirror_*(*λ*)]/{2∙[*I_cap_*(*λ*)∙*I_mirror_*(*λ*)]^1/2^} (7)

**Figure 4:**
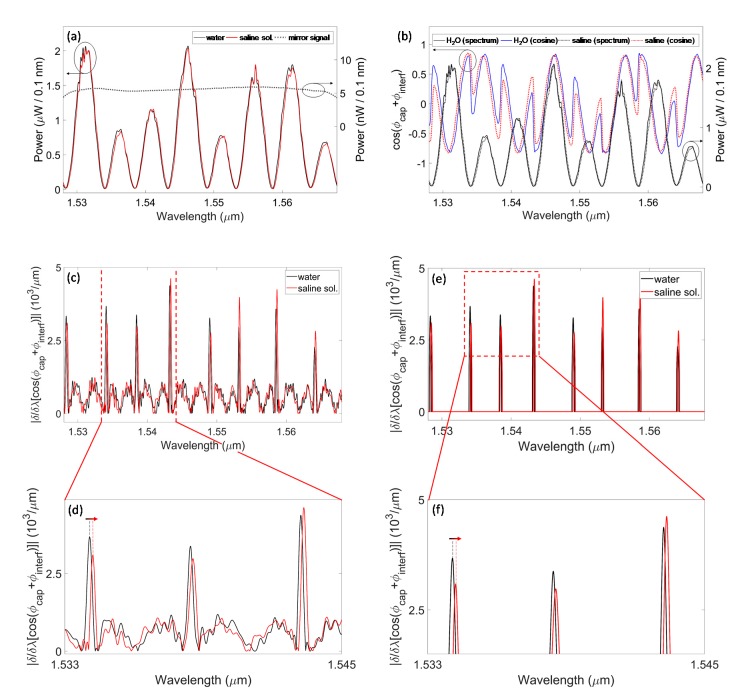
Experimental results of spectral phase-shift interferometry applied to a micro-capillary with *t_f_* = *d* = *t_b_* = 50 μm to distinguish the refractive indices of water and saline solution. (**a**) Typical signals acquired during the experiments: power spectra reflected by the capillary when it is filled with water (black solid trace) and saline solution (red solid trace) and power spectrum reflected from the reference mirror (black dotted trace), with approximately constant density over the spectral range of interest. (**b**) Cosine signals (after digital low-pass filtering) obtained with phase-sensitive detection when the capillary is filled with water (blue solid trace) and saline solution (red dotted trace): steep phase jumps are observed at the same wavelengths where minima of the reflected power spectrum occur (black solid trace: spectral amplitude results obtained with water as filling fluid; black dotted trace: same kind of signal but with saline solution). (**c**) Absolute value of the derivatives of the cosine signals with respect to the wavelength: following the shift of the peaks it is possible to retrieve the refractive index (RI) variation of the sample from water (black trace) to saline solution (red trace). (**d**) Zoomed view of (**c**) of a narrower wavelength range. The arrow highlights the spectral shift. (**e**) Absolute value of the derivatives shown in (**c**) after thresholding to better highlight the sharp peaks (black trace: water; red trace: saline solution). (**f**) Zoomed view of (**e**) of a narrower wavelength range. The arrow highlights the spectral shift. The resolution bandwidth of the OSA employed for all measurements is *RB* = 0.1 nm.

Experiments were carried out by filling the capillary channel first with distilled water and then with saline solution (Alcon Laboratories, Inc., Fort Worth, TX, USA). The RIs of these samples, measured at room temperature by means of an Abbe refractometer at a wavelength of 566 nm, were found to be *n_H2O_* = 1.3340 ± 0.0001 and *n_Saline_* = 1.3345 ± 0.0001, thus the RI difference was *Δn_H2O Saline_* = 5.5 ∙ 10^−4^ refractive index unit (RIU). These values were employed also in the theoretical analysis. Even if, in the NIR region, the absolute RI values are not exactly the same as in the visible region, their RI difference is very likely of the same order of magnitude. Figure 4a reports the acquired power spectra that are reflected by the capillary, *I_cap_*(*λ)*, when filled with water (black solid trace) and saline solution (red solid trace). The red trace is slightly red-shifted with respect to the black trace, as expected, due to the higher RI of saline solution. The line-shapes of the experimental power spectra are in qualitative agreement with the theoretical behavior, shown in Figure 2a, in terms of the number of minima and maxima in the selected span and the sequences of peaks with different amplitudes. Discrepancies are due to the effective values of the capillary dimensions, very likely not coincident with the nominal values considered in the simulations, due to fabrication tolerances. The black dotted trace in Figure 4a illustrates the power spectrum reflected from the mirror, *I_mirror_*(*λ*), with approximately constant power density over the wavelength range of interest. 

Figure 4b reports the interferometric cosine-shaped signals as a function of the wavelength obtained with phase-sensitive detection, and retrieved by applying Equation 7 to the acquired data, when the capillary is filled with water (blue solid trace) and saline solution (red dotted trace). All cosine signals were processed with a digital low-pass filter to get rid of spurious spectral ripple, very likely coming from unwanted internal reflections. The applied filter, implemented in MATLAB code, is an infinite impulse response (IIR) Butterworth low-pass filter with a cut-off *λ*^−1^ at 3000 µm^−1^ and an order of 10. Steep amplitude variations due to phase jumps are observed at the same wavelengths where resonances occur, corresponding to minima in the power spectrum reflected by the capillary. In Figure 4b, the black solid trace is the reflected power spectrum obtained with amplitude detection when water is the filling fluid, whereas the black dotted trace is the same signal for the saline-filled capillary. Moreover, the interferometric cosine signal related to saline solution is red-shifted with respect to the same kind of signal achieved with water filling. 

By calculating the derivatives of the cosine signals with respect to the wavelength and computing their absolute value (Figure 4c), the steep amplitude variations due to phase jumps are strongly enhanced, appearing as sharp peaks as a function of the wavelength. In Figure 4d, the spectral shift can be appreciated with higher magnification. The FWHMs of the three peaks are 210, 260 and 230 pm, respectively; these values are of the same magnitude as the theoretical value obtained for data simulated after adding the noise. The graphs in Figure 4c actually resemble what was calculated after noise addition and was shown in Figure 2f. In order to better highlight the derivative maxima, the amplitude threshold was set to 2200 μm^−1^ and all the signal components below this value were zeroed; results of this processing step are reported in Figure 4e and in the zoomed view of Figure 4f.

Table 1 reports the comparison between the detected wavelength position of a spectral minimum and of the correspondent derivative peak, for both water and saline solution. Average values calculated on three acquisitions and their relative standard deviations are reported. The average values of the resonance wavelength positions are the same when looking at the spectral minimum or at the derivative peak, while the standard deviation values are of the same order of magnitude. The sensitivity *S* of the selected resonance, expressed as induced wavelength shift *Δλ*, for a given RI variation *Δn* can be obtained as *S* = *Δλ*/*Δn* = 0.2 nm/(5.5 ∙ 10^−4^) RIU ≈ 360 nm/RIU. Theoretical sensitivity values quantifying the shift efficiency of resonances in the same wavelength range were found to be between 290 and 400 nm/RIU. Furthermore, considering the parameter *K_Ave_* = *Δλ_H2O Saline_*/*FSR*, where *FSR*, the free spectral range, is the wavelength separation between two consecutive minima of the same spectrum, its average value calculated for all minima in the considered spectral range is *K_Ave_* = 0.02 of the same order of magnitude of the expected theoretical value.

As mentioned in previous works, refractive index variations in a fluid sample, e.g., a solution, with respect to the reference fluid (usually the solvent) can be detected without ambiguity only if the induced spectral shift is narrower than the wavelength separation between two consecutive minima, (previously mentioned as *FSR*). When comparing saline solution to water, the RI variation induces a shift that is much smaller than the separation between consecutive minima. 

To further investigate the potentiality of spectral phase-shift interferometry for RI monitoring, experimental analyses were repeated by filling the capillary with two alcohols, ethanol and isopropanol. The RIs of these samples, measured at room temperature by means of an Abbe refractometer at a wavelength of 566 nm, were found to be *n_ETOH_* = 1.3640 ± 0.0001 and *n_ISOP_* = 1.3772 ± 0.0001, thus, the RI difference was *Δn_Alcohols_* = 13.2 ∙ 10^-3^ RIU. Considering a sensitivity of the order of 360 nm/RIU, previously calculated from the experimental results attained with water and saline solutions, when substituting ethanol with isopropanol we expect a spectral shift of the order of 5 nm—almost as large as the typical wavelength separation between consecutive minima relative to resonances of the same order. Note that it was already demonstrated in previous works [17] that the separation between consecutive minima differs from one to another. This second set of experiments was performed to demonstrate that phase-sensitive detection was feasible, and also suitable, for large RI variations, close to the limit of the unambiguous range.

**Figure 5:**
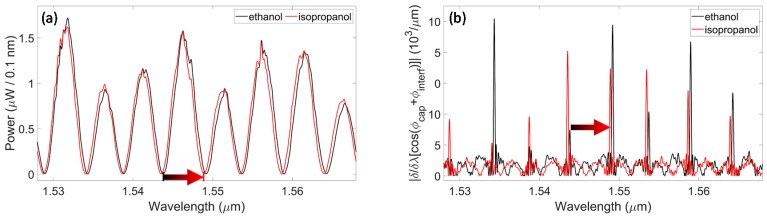
Experimental results for a micro-capillary with *t_f_* = *d* = *t_b_* = 50 μm obtained by filling the channel with ethanol and isopropanol. (**a**) Typical power spectra reflected by the capillary filled with ethanol (black trace) and isopropanol (red trace). The arrow highlights the induced spectral shift. (**b**) Absolute value of the derivatives of the cosine signals with respect to the wavelength (black trace: ethanol; red trace: isopropanol).

Data were collected by first filling the capillary with ethanol and then with isopropanol. We followed the same procedure and acquired the same sequence of data previously described for the experiment with water and saline solution. For the sake of brevity, we report here only the most significant results. Figure 5a reports the power spectra reflected from the capillary itself, thus obtained with the amplitude detection, when the channel is filled with ethanol (black trace) and isopropanol (red trace). The absolute values of the derivatives of the cosine signals (as a function of the wavelength), obtained with phase-sensitive detection, are shown in Figure 5b; in agreement with previous results, sharp peaks are located at the same wavelength positions as the reflectivity minima. As expected, the peaks undergo a wide red-shift, almost as large as the separation between consecutive resonances, when ethanol is substituted by isopropanol in the capillary channel. In this case, the wavelength shift of the resonance indicated by the arrow is *Δλ_ISOP_-_ETOH_* = 5.15 nm. Moreover, we experimentally obtained *K_Ave_* = *Δλ_ISOP_-_ETOH_*/FSR = 0.95, in agreement with the theoretical calculation.

## 7. Conclusions

In this work, we presented an innovative optical method for detecting the RI of fluid samples based on the phase detection of the optical resonances of rectangular glass micro-capillaries. By inserting the device at the end of the measurement arm of a Michelson interferometer and using a broadband light source, it was possible to detect the wavelength position of the resonances that correspond to the sharp phase jumps of the interferometric cosine signal. Moreover, it was demonstrated that the wavelength positions at which the phase variations occur shift towards higher values when the RI of the sample filling the capillary channel increases. First, the theoretical sensor response was investigated by modelling a capillary with geometrical dimensions *t_f_* = *d* = *t_b_* = 50 μm as an optical resonator and retrieving the theoretical interferometric cosine signals. In particular, the phase jumps can be better highlighted by computing the absolute value of the derivative of the cosine signals with respect to the wavelength. Then, experimental analyses were performed by filling the capillary channel with water and saline solution (*Δn_H2O Saline_* = 5.5 ∙ 10^−4^ RIU), to prove the capability of the sensor to discriminate among these kinds of samples. The results are in good agreement with the theoretical analysis. The same experiments were repeated by inserting ethanol and isopropanol with a larger RI variation (Δ*n_Alcohols_* = 13.2 ∙ 10^−3^ RIU) into the channel; the sensor dynamic range is wide enough to ensure RI monitoring in this situation as well. It is thus feasible, combining the interferometric setup and micro-fluidic glass platform, to perform innovative phase-based measurements of the RI of ultra-low volumes of liquids. The main advantage of the interferometric method over the spectral amplitude readout consists in measuring the wavelength position of narrow and well-defined maxima of the cosine signal derivative, instead of broad minima of the capillary reflection spectrum. Moreover, minima are more sensitive than maxima to amplitude oscillations due to the detection of noise or fluctuations in the source-emitted power that could even be wavelength dependent. Therefore, the interferometric method is less affected by all these spurious fluctuations. Furthermore, even if the sensitivity *S* = *Δλ*/*Δn* is comparable for both detection techniques, because it is an intrinsic feature of the transduction method based on resonance shift, the spectral phase technique could provide better performances. Indeed, in the neighborhood of a resonance, we can define a responsivity (*R*) for both measurement methods given, for phase interferometric readout, by *R_interferometric_* = *Δ*cos(*φ_tot_*(*λ*))/*Δn* = [*Δ*cos(*φ_tot_*(*λ*))/*Δλ*]∙*S* and, for spectral amplitude readout, by *R_spectral_* = *ΔI_cap_*(*λ*)/*Δn =* [*ΔI_cap_*(*λ*)/*Δλ*]∙*S*. As is evident, *R_interferometric_* is much higher than *R_spectral_*. Finally, it is also underlined in the literature that the performance is better when using phase-sensitive detection [24]. Future work will be devoted to the investigation of a more compact instrumental setup that does not require the use of the OSA. By sweeping the emission wavelength of a semiconductor laser across an optical resonance and simultaneously introducing a higher frequency amplitude modulation, phase detection of the resonance shift induced by small refractive index variations could be carried out with a photodiode outside the baseband.

## Figures and Tables

**Table 1:** Comparison between the results of phase-sensitive detection and spectral amplitude detection.

		Average Value [nm]	Standard Deviation [nm]
**water**	spectrum minimum	1533.7	24.1
	derivative peak	1533.7	20.7
**Saline** **solution**	spectrum minimum	1533.9	17.3
	derivative peak	1533.7	72.6
